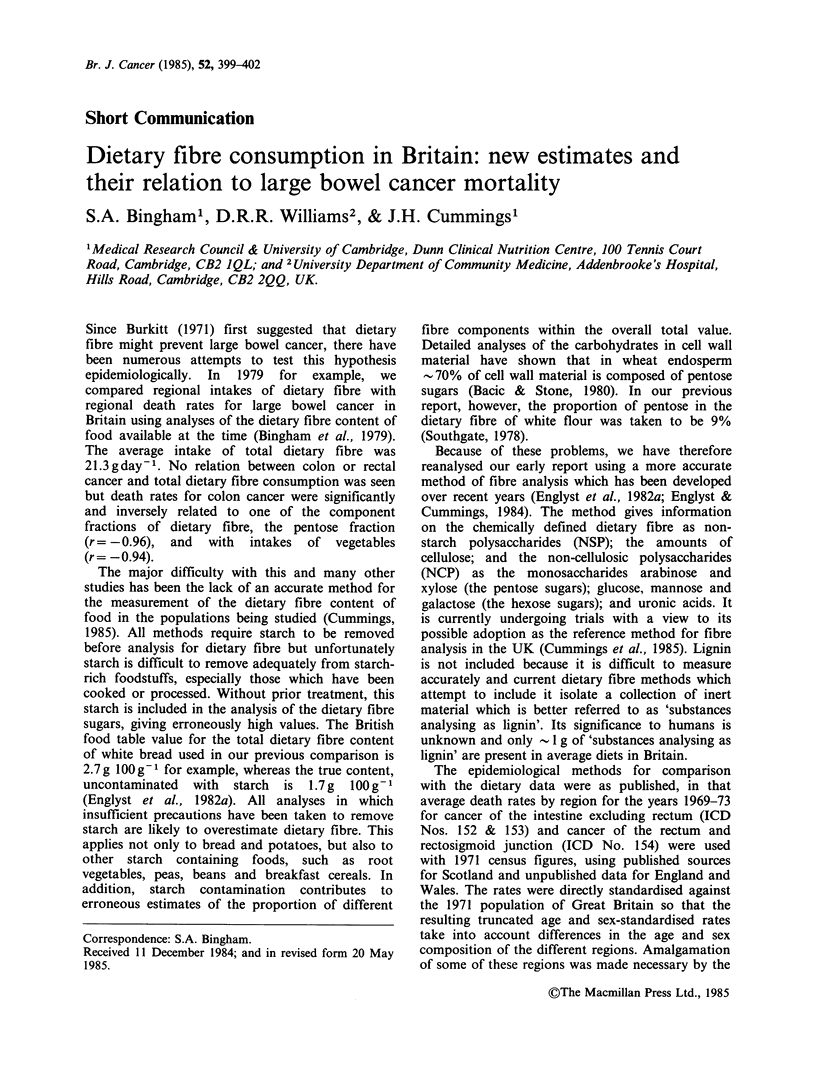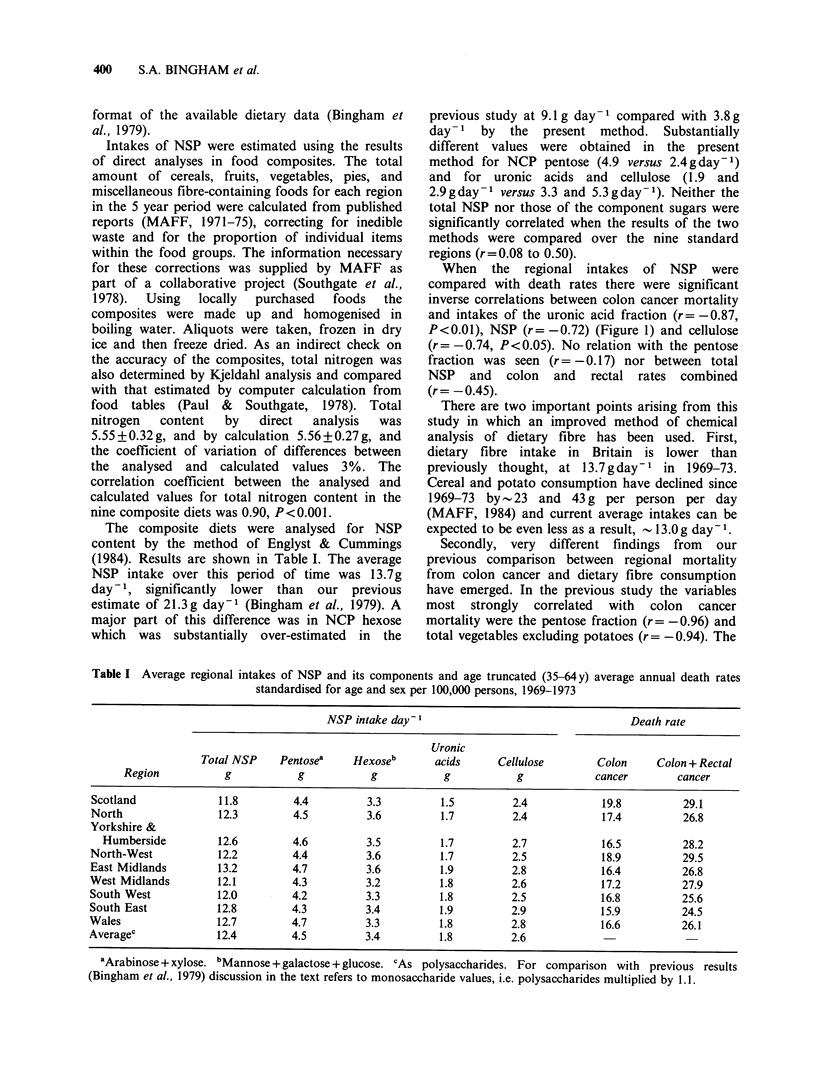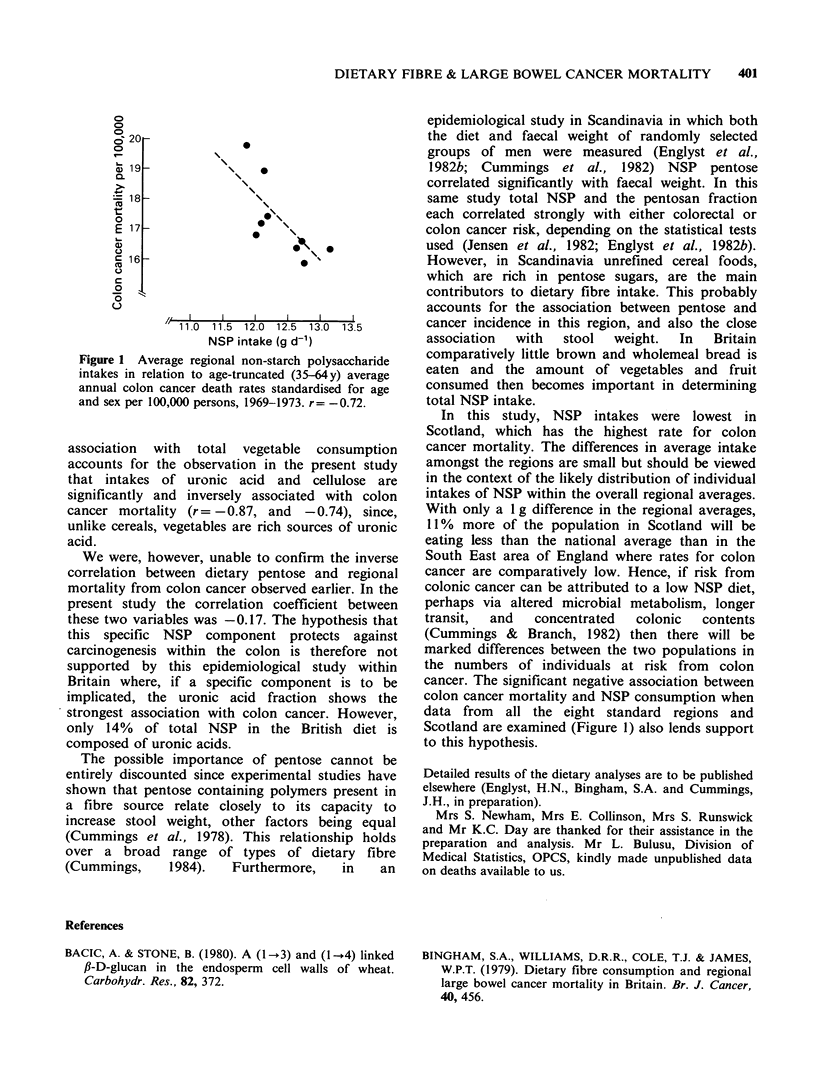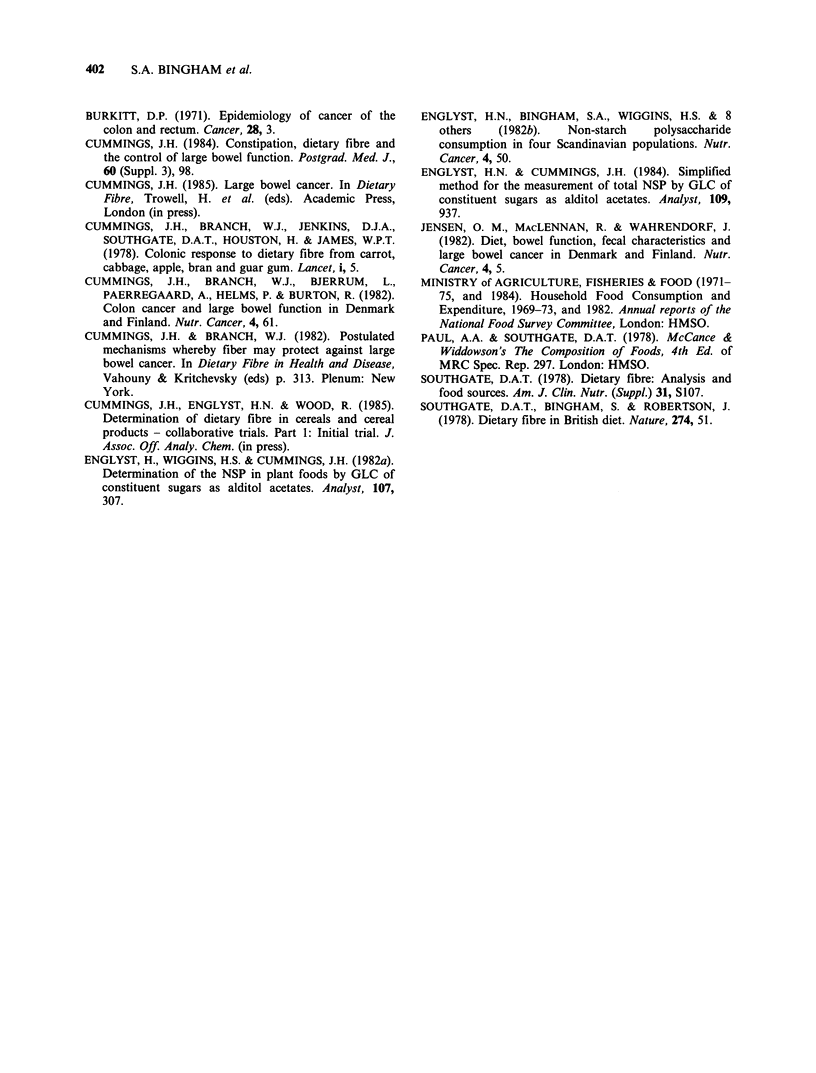# Dietary fibre consumption in Britain: new estimates and their relation to large bowel cancer mortality.

**DOI:** 10.1038/bjc.1985.207

**Published:** 1985-09

**Authors:** S. A. Bingham, D. R. Williams, J. H. Cummings


*Br. J. Cancer* (1985), **52**, 399–402

### Short Communication

### Dietary fibre consumption in Britain: new estimates and their relation to large bowel cancer mortality

S.A. Bingham[1], D.R.R. Williams[2], & J.H. Cummings[1]

[1]*Medical Research Council & University of Cambridge, Dunn Clinical Nutrition Centre, 100 Tennis Court Road, Cambridge, CB2 1QL; and* [2]*University Department of Community Medicine, Addenbrooke's Hospital, Hills Road, Cambridge, CB2 2QQ, UK.*

Since Burkitt (1971) first suggested that dietary fibre might prevent large bowel cancer, there have been numerous attempts to test this hypothesis epidemiologically. In 1979 for example, we compared regional intakes of dietary fibre with regional death rates for large bowel cancer in Britain using analyses of the dietary fibre content of food available at the time (Bingham *et al.*, 1979). The average intake of total dietary fibre was 21.3 g day$^{-1}$. No relation between colon or rectal cancer and total dietary fibre consumption was seen but death rates for colon cancer were significantly and inversely related to one of the component fractions of dietary fibre, the pentose fraction ($r=-0.96$), and with intakes of vegetables ($r=-0.94$).

The major difficulty with this and many other studies has been the lack of an accurate method for the measurement of the dietary fibre content of food in the populations being studied (Cummings, 1985). All methods require starch to be removed before analysis for dietary fibre but unfortunately starch is difficult to remove adequately from starch-rich foodstuffs, especially those which have been cooked or processed. Without prior treatment, this starch is included in the analysis of the dietary fibre sugars, giving erroneously high values. The British food table value for the total dietary fibre content of white bread used in our previous comparison is 2.7 g 100 g$^{-1}$ for example, whereas the true content, uncontaminated with starch is 1.7 g 100 g$^{-1}$ (Englyst *et al.*, 1982*a*). All analyses in which insufficient precautions have been taken to remove starch are likely to overestimate dietary fibre. This applies not only to bread and potatoes, but also to other starch containing foods, such as root vegetables, peas, beans and breakfast cereals. In addition, starch contamination contributes to erroneous estimates of the proportion of different fibre components within the overall total value. Detailed analyses of the carbohydrates in cell wall material have shown that in wheat endosperm ~70% of cell wall material is composed of pentose sugars (Bacic & Stone, 1980). In our previous report, however, the proportion of pentose in the dietary fibre of white flour was taken to be 9% (Southgate, 1978).

Because of these problems, we have therefore reanalysed our early report using a more accurate method of fibre analysis which has been developed over recent years (Englyst *et al.*, 1982*a*; Englyst & Cummings, 1984). The method gives information on the chemically defined dietary fibre as non-starch polysaccharides (NSP); the amounts of cellulose; and the non-cellulosic polysaccharides (NCP) as the monosaccharides arabinose and xylose (the pentose sugars); glucose, mannose and galactose (the hexose sugars); and uronic acids. It is currently undergoing trials with a view to its possible adoption as the reference method for fibre analysis in the UK (Cummings *et al.*, 1985). Lignin is not included because it is difficult to measure accurately and current dietary fibre methods which attempt to include it isolate a collection of inert material which is better referred to as 'substances analysing as lignin'. Its significance to humans is unknown and only ~1 g of 'substances analysing as lignin' are present in average diets in Britain.

The epidemiological methods for comparison with the dietary data were as published, in that average death rates by region for the years 1969–73 for cancer of the intestine excluding rectum (ICD Nos. 152 & 153) and cancer of the rectum and rectosigmoid junction (ICD No. 154) were used with 1971 census figures, using published sources for Scotland and unpublished data for England and Wales. The rates were directly standardised against the 1971 population of Great Britain so that the resulting truncated age and sex-standardised rates take into account differences in the age and sex composition of the different regions. Amalgamation of some of these regions was made necessary by the

Correspondence: S.A. Bingham.
Received 11 December 1984; and in revised form 20 May 1985.

©The Macmillan Press Ltd., 1985

format of the available dietary data (Bingham *et al.*, 1979).

Intakes of NSP were estimated using the results of direct analyses in food composites. The total amount of cereals, fruits, vegetables, pies, and miscellaneous fibre-containing foods for each region in the 5 year period were calculated from published reports (MAFF, 1971–75), correcting for inedible waste and for the proportion of individual items within the food groups. The information necessary for these corrections was supplied by MAFF as part of a collaborative project (Southgate *et al.*, 1978). Using locally purchased foods the composites were made up and homogenised in boiling water. Aliquots were taken, frozen in dry ice and then freeze dried. As an indirect check on the accuracy of the composites, total nitrogen was also determined by Kjeldahl analysis and compared with that estimated by computer calculation from food tables (Paul & Southgate, 1978). Total nitrogen content by direct analysis was $5.55 \pm 0.32$ g, and by calculation $5.56 \pm 0.27$ g, and the coefficient of variation of differences between the analysed and calculated values 3%. The correlation coefficient between the analysed and calculated values for total nitrogen content in the nine composite diets was 0.90, $P<0.001$.

The composite diets were analysed for NSP content by the method of Englyst & Cummings (1984). Results are shown in Table I. The average NSP intake over this period of time was 13.7g $day^{-1}$, significantly lower than our previous estimate of 21.3 g $day^{-1}$ (Bingham *et al.*, 1979). A major part of this difference was in NCP hexose which was substantially over-estimated in the previous study at 9.1 g $day^{-1}$ compared with 3.8 g $day^{-1}$ by the present method. Substantially different values were obtained in the present method for NCP pentose (4.9 *versus* 2.4 g $day^{-1}$) and for uronic acids and cellulose (1.9 and 2.9 g $day^{-1}$ *versus* 3.3 and 5.3 g $day^{-1}$). Neither the total NSP nor those of the component sugars were significantly correlated when the results of the two methods were compared over the nine standard regions ($r=0.08$ to 0.50).

When the regional intakes of NSP were compared with death rates there were significant inverse correlations between colon cancer mortality and intakes of the uronic acid fraction ($r=-0.87$, $P<0.01$), NSP ($r=-0.72$) (Figure 1) and cellulose ($r=-0.74$, $P<0.05$). No relation with the pentose fraction was seen ($r=-0.17$) nor between total NSP and colon and rectal rates combined ($r=-0.45$).

There are two important points arising from this study in which an improved method of chemical analysis of dietary fibre has been used. First, dietary fibre intake in Britain is lower than previously thought, at 13.7 g $day^{-1}$ in 1969–73. Cereal and potato consumption have declined since 1969–73 by ~23 and 43 g per person per day (MAFF, 1984) and current average intakes can be expected to be even less as a result, ~13.0 g $day^{-1}$.

Secondly, very different findings from our previous comparison between regional mortality from colon cancer and dietary fibre consumption have emerged. In the previous study the variables most strongly correlated with colon cancer mortality were the pentose fraction ($r=-0.96$) and total vegetables excluding potatoes ($r=-0.94$). The

**Table I** Average regional intakes of NSP and its components and age truncated (35–64 y) average annual death rates standardised for age and sex per 100,000 persons, 1969–1973

| Region | *NSP intake day$^{-1}$* Total NSP g | Pentose[a] g | Hexose[b] g | Uronic acids g | Cellulose g | *Death rate* Colon cancer | Colon + Rectal cancer |
|---|---|---|---|---|---|---|---|
| Scotland | 11.8 | 4.4 | 3.3 | 1.5 | 2.4 | 19.8 | 29.1 |
| North | 12.3 | 4.5 | 3.6 | 1.7 | 2.4 | 17.4 | 26.8 |
| Yorkshire & Humberside | 12.6 | 4.6 | 3.5 | 1.7 | 2.7 | 16.5 | 28.2 |
| North-West | 12.2 | 4.4 | 3.6 | 1.7 | 2.5 | 18.9 | 29.5 |
| East Midlands | 13.2 | 4.7 | 3.6 | 1.9 | 2.8 | 16.4 | 26.8 |
| West Midlands | 12.1 | 4.3 | 3.2 | 1.8 | 2.6 | 17.2 | 27.9 |
| South West | 12.0 | 4.2 | 3.3 | 1.8 | 2.5 | 16.8 | 25.6 |
| South East | 12.8 | 4.3 | 3.4 | 1.9 | 2.9 | 15.9 | 24.5 |
| Wales | 12.7 | 4.7 | 3.3 | 1.8 | 2.8 | 16.6 | 26.1 |
| Average[c] | 12.4 | 4.5 | 3.4 | 1.8 | 2.6 | — | — |

[a]Arabinose + xylose. [b]Mannose + galactose + glucose. [c]As polysaccharides. For comparison with previous results (Bingham *et al.*, 1979) discussion in the text refers to monosaccharide values, i.e. polysaccharides multiplied by 1.1.

**Figure 1** Average regional non-starch polysaccharide intakes in relation to age-truncated (35–64 y) average annual colon cancer death rates standardised for age and sex per 100,000 persons, 1969–1973. $r = -0.72$.

association with total vegetable consumption accounts for the observation in the present study that intakes of uronic acid and cellulose are significantly and inversely associated with colon cancer mortality ($r = -0.87$, and $-0.74$), since, unlike cereals, vegetables are rich sources of uronic acid.

We were, however, unable to confirm the inverse correlation between dietary pentose and regional mortality from colon cancer observed earlier. In the present study the correlation coefficient between these two variables was $-0.17$. The hypothesis that this specific NSP component protects against carcinogenesis within the colon is therefore not supported by this epidemiological study within Britain where, if a specific component is to be implicated, the uronic acid fraction shows the strongest association with colon cancer. However, only 14% of total NSP in the British diet is composed of uronic acids.

The possible importance of pentose cannot be entirely discounted since experimental studies have shown that pentose containing polymers present in a fibre source relate closely to its capacity to increase stool weight, other factors being equal (Cummings *et al.*, 1978). This relationship holds over a broad range of types of dietary fibre (Cummings, 1984). Furthermore, in an epidemiological study in Scandinavia in which both the diet and faecal weight of randomly selected groups of men were measured (Englyst *et al.*, 1982*b*; Cummings *et al.*, 1982) NSP pentose correlated significantly with faecal weight. In this same study total NSP and the pentosan fraction each correlated strongly with either colorectal or colon cancer risk, depending on the statistical tests used (Jensen *et al.*, 1982; Englyst *et al.*, 1982*b*). However, in Scandinavia unrefined cereal foods, which are rich in pentose sugars, are the main contributors to dietary fibre intake. This probably accounts for the association between pentose and cancer incidence in this region, and also the close association with stool weight. In Britain comparatively little brown and wholemeal bread is eaten and the amount of vegetables and fruit consumed then becomes important in determining total NSP intake.

In this study, NSP intakes were lowest in Scotland, which has the highest rate for colon cancer mortality. The differences in average intake amongst the regions are small but should be viewed in the context of the likely distribution of individual intakes of NSP within the overall regional averages. With only a 1 g difference in the regional averages, 11% more of the population in Scotland will be eating less than the national average than in the South East area of England where rates for colon cancer are comparatively low. Hence, if risk from colonic cancer can be attributed to a low NSP diet, perhaps via altered microbial metabolism, longer transit, and concentrated colonic contents (Cummings & Branch, 1982) then there will be marked differences between the two populations in the numbers of individuals at risk from colon cancer. The significant negative association between colon cancer mortality and NSP consumption when data from all the eight standard regions and Scotland are examined (Figure 1) also lends support to this hypothesis.

Detailed results of the dietary analyses are to be published elsewhere (Englyst, H.N., Bingham, S.A. and Cummings, J.H., in preparation).

Mrs S. Newham, Mrs E. Collinson, Mrs S. Runswick and Mr K.C. Day are thanked for their assistance in the preparation and analysis. Mr L. Bulusu, Division of Medical Statistics, OPCS, kindly made unpublished data on deaths available to us.

### References

BACIC, A. & STONE, B. (1980). A (1→3) and (1→4) linked $\beta$-D-glucan in the endosperm cell walls of wheat. *Carbohydr. Res.*, **82**, 372.

BINGHAM, S.A., WILLIAMS, D.R.R., COLE, T.J. & JAMES, W.P.T. (1979). Dietary fibre consumption and regional large bowel cancer mortality in Britain. *Br. J. Cancer*, **40**, 456.

BURKITT, D.P. (1971). Epidemiology of cancer of the colon and rectum. *Cancer*, **28**, 3.

CUMMINGS, J.H. (1984). Constipation, dietary fibre and the control of large bowel function. *Postgrad. Med. J.*, **60** (Suppl. 3), 98.

CUMMINGS, J.H. (1985). Large bowel cancer. In *Dietary Fibre*, Trowell, H. *et al.* (eds). Academic Press, London (in press).

CUMMINGS, J.H., BRANCH, W.J., JENKINS, D.J.A., SOUTHGATE, D.A.T., HOUSTON, H. & JAMES, W.P.T. (1978). Colonic response to dietary fibre from carrot, cabbage, apple, bran and guar gum. *Lancet*, **i**, 5.

CUMMINGS, J.H., BRANCH, W.J., BJERRUM, L., PAERREGAARD, A., HELMS, P. & BURTON, R. (1982). Colon cancer and large bowel function in Denmark and Finland. *Nutr. Cancer*, **4**, 61.

CUMMINGS, J.H. & BRANCH, W.J. (1982). Postulated mechanisms whereby fiber may protect against large bowel cancer. In *Dietary Fibre in Health and Disease*, Vahouny & Kritchevsky (eds) p. 313. Plenum: New York.

CUMMINGS, J.H., ENGLYST, H.N. & WOOD, R. (1985). Determination of dietary fibre in cereals and cereal products – collaborative trials. Part 1: Initial trial. *J. Assoc. Off. Analy. Chem.* (in press).

ENGLYST, H., WIGGINS, H.S. & CUMMINGS, J.H. (1982*a*). Determination of the NSP in plant foods by GLC of constituent sugars as alditol acetates. *Analyst*, **107**, 307.

ENGLYST, H.N., BINGHAM, S.A., WIGGINS, H.S. & 8 others (1982*b*). Non-starch polysaccharide consumption in four Scandinavian populations. *Nutr. Cancer*, **4**, 50.

ENGLYST, H.N. & CUMMINGS, J.H. (1984). Simplified method for the measurement of total NSP by GLC of constituent sugars as alditol acetates. *Analyst*, **109**, 937.

JENSEN, O. M., MACLENNAN, R. & WAHRENDORF, J. (1982). Diet, bowel function, fecal characteristics and large bowel cancer in Denmark and Finland. *Nutr. Cancer*, **4**, 5.

MINISTRY of AGRICULTURE, FISHERIES & FOOD (1971–75, and 1984). Household Food Consumption and Expenditure, 1969–73, and 1982. *Annual reports of the National Food Survey Committee*, London: HMSO.

PAUL, A.A. & SOUTHGATE, D.A.T. (1978). *McCance & Widdowson's The Composition of Foods, 4th Ed.* of MRC Spec. Rep. 297. London: HMSO.

SOUTHGATE, D.A.T. (1978). Dietary fibre: Analysis and food sources. *Am. J. Clin. Nutr.* (*Suppl.*) **31**, S107.

SOUTHGATE, D.A.T., BINGHAM, S. & ROBERTSON, J. (1978). Dietary fibre in British diet. *Nature*, **274**, 51.